# Temporal and spatial pattern of DNA damage in neurons following spinal cord Injury in mice

**DOI:** 10.1186/s12929-024-01104-8

**Published:** 2025-01-23

**Authors:** Elle EM Scheijen, Naomi Veeningen, Sam Duwé, Anna Ivanova, Jana Van Broeckhoven, Sven Hendrix, David M Wilson

**Affiliations:** 1 Neurosciences, Biomedical Research Institute, Hasselt University, Agoralaan Building C, 3590 Diepenbeek, Belgium; 2 Immunology and Infection, Biomedical Research Institute, Hasselt University, Agoralaan Building C, 3590 Diepenbeek, Belgium; 3 Advanced Optical Microscopy Centre, Biomedical Research Institute, Hasselt University, Agoralaan Building C, 3590 Diepenbeek, Belgium; 4 Data Science Institute, Biomedical Research Institute, Hasselt University, Agoralaan Building D, 3590 Diepenbeek, Belgium; 5 Institute for Translational Medicine, Medical School Hamburg, Am Kaiserkai 1, 20457 Hamburg, Germany

**Keywords:** Spinal cord injury, DNA damage, Oxidative stress, Gamma-H2AX, Neuronal death, DNA repair

## Abstract

Deficient DNA repair and excessive DNA damage contribute to neurodegenerative disease. However, the role of DNA damage and repair in spinal cord injury (SCI) is unclear. SCI, a debilitating disruption of the structural and biological network of the spinal cord, is characterized by oxidative stress. Nevertheless, the pathophysiological mechanisms leading to neuronal loss following SCI remain incompletely defined. Methods: Using a contusion model, a severe SCI was induced at the L1 spinal level in C57Bl/6J mice. The temporal and spatial presence of DNA damage was then determined via immunolabeling for the DNA damage marker, γH2AX, from 1 h post-injury (hpi) to 28 days post-injury (dpi). Results: Our analysis revealed that increased DNA damage foci were present from 1 hpi to 3 dpi in SCI mice relative to controls (sham surgery and naive), with the damage signal spreading over time longitudinally from the affected area to more rostral and caudal regions. Co-labeling of γH2AX with NeuN revealed neuronal specificity of DNA damage, with increased early cell death (pan-nuclear γH2AX) peaking at 1 dpi and apoptosis (cleaved Caspase-3) arising later at 3 dpi. Conclusion: Our study indicates a possible role of DNA damage in neuronal loss following SCI and highlights the need for early interventions targeting DNA repair to preserve neuronal tissue.

**Supplementary Information:**

The online version contains supplementary material available at 10.1186/s12929-024-01104-8.

## Introduction

Spinal cord injury (SCI) is a severely debilitating disorder caused by a traumatic or non-traumatic primary injury, such as a fall, accident, assault, nearby tumor, vascular condition, infection, or birth defect. The initial SCI subsequently evolves into a secondary injury (a.k.a., phase) characterized by spinal shock, vascular dysfunction, cell membrane hyperpermeability, excitotoxicity, neuroinflammation, glial scar formation, activation of Nogo receptors, and oxidative stress [1]. This secondary phase can be divided into different stages, namely acute (0–2 dpi), sub-acute (3–14 dpi), and early chronic (from 2 weeks on) [2]. The above events collectively cause the gradual deterioration of the central nervous system (CNS) network, leading to reduced physical capacity, psychological health, and overall quality of life.

Due to resulting mitochondrial dysfunction and hyperinflammatory responses, reactive oxygen species (ROS) are excessively present at the injury site [3]. With only minimal endogenous antioxidants available within the CNS, oxidative stress is presumably a significant cause of the secondary neuronal cell loss seen following an SCI [4]. Consistently, several reports have documented increased markers of oxidative stress, and antioxidant therapies have shown some promise in mouse SCI [5–9]. Notably, ROS can attack and cause damage to all macromolecules, such as proteins, lipids, RNA, and DNA. While most of these damaged molecules can be removed and replaced by cellular turnover mechanisms, damaged DNA, especially in terminally differentiated cells such as post-mitotic neurons, must be resolved and restored to its original state to avoid cell death or network dysfunction [10].

Excessive DNA damage is an important contributor to several neurodegenerative diseases, e.g., Alzheimer disease, Parkinson disease, Charcot-Marie-Tooth disease, Huntington disease, and amyotrophic lateral sclerosis [11, 12]. In particular, DNA damage poses a severe threat to neurons, as persistent lesions can alter gene expression landscapes, introduce errors in the genetic code, or interfere with normal DNA metabolic events, such as transcription [13]. If DNA damage is excessively present due to increased genotoxic exposure or insufficient DNA repair capacity, neurons will be instructed to undergo programmed cell death or cellular senescence. It is, therefore, reasonable to speculate that oxidative stress-induced DNA damage is responsible for the neuronal loss and dysfunction seen in SCI pathophysiology. Multiple types of DNA damage can be generated during oxidative stress. For example, a wide range of base modifications, e.g., 8-oxoguanine, as well as apurinic/apyrimidinic sites (AP sites), can be produced by direct attack of ROS of DNA. DNA strand breaks, both single-strand breaks (SSBs) and double-strand breaks (DSBs), can also occur due to ROS attack of the sugar-phosphodiester backbone [14]. Other oxidative DNA modifications include intra- and interstrand crosslinks and protein-DNA adducts, with most eliciting harmful, often lethal, downstream consequences.

Apoptosis-related DNA fragmentation was already established as part of SCI’s pathophysiology in 1997 [15]. However, whether reversible DNA damage that can be therapeutically targeted contributes to the pathophysiology of SCI remains unclear. In our recent review [16], we show that studies regarding DNA damage or protective DNA damage/repair responses in SCI are limited and often report conflicting results regarding the presence, timing, and magnitude of DNA damage, partly due to inappropriate controls or other experimental limitations. Moreover, no information is currently available regarding cell-type-specific effects [16], emphasizing the need for further investigations to clarify the scope and patterns of DNA damage after an SCI. This study elucidates the temporal, spatial, and neuron-specific hallmarks of DNA damage in a mouse SCI contusion model. Our analysis shows that DNA damage occurs primarily at early time points (up to 3 days post-injury) and spreads longitudinally outwards from the lesion center. DNA damage is present in neurons and precedes neuronal cell death, indicating the importance of starting treatments early to prevent neuronal loss.

## Materials and methods

### Animals

Female C57Bl/6J mice (Janvier Labs, 10 weeks, 20 g ± 1.5 g) were purchased and housed according to general guidelines on protecting animals used for scientific purposes (EU Directives 201/63/EU). Before experimental use, the mice could acclimate for two weeks under standard housing conditions, i.e., 12 h day/night light cycle, 20–24 °C, 40–60% room humidity, food and water *ad libitum*. The Ethical Committee for Animal Experiments of Hasselt University approved the animal experiments.

## Contusion spinal cord injury

Two independent in vivo studies were performed with a total of *n* = 10 per group. 12 week-old mice were anesthetized using 2–3% isoflurane (IsoFlo). A partial laminectomy of spinal cord level lumbar 1 (L1) exposed the spinal cord for contusion, which was induced using the Infinite Horizon Impactor (Precision Systems and Instrumentation Impactors, U.S.A). A 75 kdyne force was applied at the center of spinal L1 without dwell time. The back muscles were sutured, and the skin was closed using wound clips (Autoclip). Postoperative, the mice received buprenorphine (0.1 mg/kg, subcutaneous) and glucose (20%, intraperitoneally). The mice recovered in a temperature-controlled incubator (32–34 °C) until they regained consciousness. Due to dropout, the final number of animals within the SCI groups ranges from *n* = 6–10. Sham surgery (sham) mice were included as controls. They received the same surgery but without the spinal contusion. Naive mice were included to assess the effects of surgery.

## Postoperative follow-up of animals

The mice were checked daily for the first 8 days and afterward every other day from day 10 to 28. During checks, the bladders were manually voided until the mice ceased to retain urine. Enrofloxacin (0.2 mg/ml) was added to the drinking water until day 7 to avoid urinary tract infections. The Basso Mouse Scale (BMS) score was taken to evaluate functional recovery post-injury at the specified time points [17].

## Immunohistochemistry

At several time points post-injury (1 h post-injury (hpi), 1 day post-injury (dpi), 3 dpi, 7 dpi, and 28 dpi), animals were sacrificed using natriumpentobarbital (2 g/kg). The mice were transcardially perfused with Ringer solution and 4% paraformaldehyde (PFA) in phosphate-buffered saline (PBS). The spinal cords were isolated via longitudinal laminectomy and post-fixated overnight in 4% PFA-5% sucrose at 4 °C. After incubation in 30% sucrose for 72 h, the spinal cords were embedded in Tissue-Tek O.C. 10 μm longitudinal cryosections were obtained on a Leica CM1900 cryostat (Leica Biosystems, Belgium).

For γH2AX, cleaved Caspase-3, and 53BP1 labeling, sections underwent antigen retrieval using 1X HistoVT One (Nacalai) solution at 65 °C for 20 min, followed by a pre-permeabilization in 2% Triton-X100 in Tris-buffered saline (TBS) for 10 min at room temperature (RT). For myelin basic protein (MBP) labeling, sections were post-fixated with ice-cold acetone for 10 min at 4 °C and air-dried. Following blocking in 10% goat serum + 5% BSA for 1 h at RT, the sections were incubated overnight at 4 °C with primary antibody (rabbit anti-γH2AX, Cell Signaling Technology 2577, 1/1000; rabbit anti-53BP1, Novus Biologicals NB 100–304, 1/1000; mouse anti-NeuN, Merck MAB377, 1/1000; rabbit anti-Cleaved Caspase-3, Cell Signaling Technology CST 9661, 1/200; rat anti-MBP, Merck MAB386, 1/500). Secondary antibodies (goat anti-rabbit-647, Invitrogen A21245, 1/800; goat anti-mouse IgG1-488, Invitrogen A21121, 1/800; goat anti-rat, Invitrogen A21247, 1/400) were applied for 1 h at RT. Counterstaining with DAPI (Thermo Fisher, 62248; 1/10 000) was performed for 10 min at RT, and slides were mounted with Fluoromount G (Invitrogen 00–4958-02).

## Image acquisition and analysis

Fluorescence images of the slides were acquired using a Zeiss Axio Scan Z.1 widefield slide scanner equipped with a Zeiss Colibri 7 R[G/Y]B-UV LED light source and a Zeiss Axiocam 506 mono (D) camera. Image acquisition was controlled using ZEN Blue 3.1. On each slide, the regions of interest containing the spinal cord sections were indicated by hand utilizing the ROI tools in ZEN and automatically focused using the DAPI signal.

Multicolor tile scan acquisitions of the entire spinal cord sections were acquired with 10% overlap between neighboring tiles using a Plan-Apochromat 20x/0.80 M27 objective lens, providing a pixel size of 0.227 μm x 0.227 μm. To minimize image size, automated Online stitching was performed with Pyramid active, jpegXR active, and lossy compression of 85%. Excitation light for DAPI, Alexa Fluor 488, and Alexa Fluor 647 was provided by the “UV”, “Blue”, and “Red” channels of the Colibri 7 LED source, respectively. Fluorophore emission was split from the excitation light and filtered using a Zeiss FSet 90 HE LED filter set. Acquisition settings, including exposure time and LED excitation intensity, were set so the detected signal used 30–40% of the full dynamic range.

Images were exported as .czi files and analyzed using QuPath 0.4.4 [18], following the workflow in Table 1. Based on visual inspection, the affected area was annotated by drawing a box that is 5000 μm wide and the full height of the spinal cord around the center of the lesion. Cell detection was performed in the DAPI or 488 channels (in the case of NeuN), depending on the experiment. Thresholds of positive cells were determined per experiment (either negative/positive or negative/foci/pan). Representative images were exported as Rendered RBG (with overlays) in TIFF. Images were rotated and provided with a scale bar in Fiji ImageJ [19]. All data points are the average of 4–7 technical replicates (sections taken 50 μm apart), calculated as percentages over total cells and normalized to the naive mice. The contrast and brightness have been adjusted per image for display and printing purposes.

**Table 1:** Image analysis workflow

Step	Action	Result
1	‘Create project’ in QuPath	NA
2	Load all images of the experiment	NA
3	Adjust ‘Brightness & contrast’ for good visualization of the markers to the eye and apply to all images	Positive cells can be easily identified by eye
4	‘Annotate’ the region of interest in each sample	The area for analysis is delineated
5	Run ‘cell detection’ with optimized parameters for the sample	All cells are identified and delineated
6	Determine the threshold(s) of the marker of interest in ‘Detection results’	Threshold(s) for each positive cell category are established
7	Run ‘Positive cell detection’ using the optimized cell detection parameters and the threshold(s) determined in step 5	Positive cells are classified into groups depending on their signal
8	Make a script including: ‘clearDetections’ ‘selectAnnotations’ ‘runPlugin’ ◊ Positive cell detection	A script for automated image analysis
9	Run the script on the complete project using ‘Run for project’	All images are analyzed
10	‘Export measurements’ as .tsv with Export type set to Annotations	A file with the analysis data
11	Perform data and statistical analysis on the data	NA

NA: not applicable

### Spatial analysis

The spatial distribution of spinal cord cells with γH2AX signal was analyzed by exporting analysis data from QuPath and loading the data in R. The x coordinate of the lesion center of each spinal cord slice was extracted, and for each cell in each slide, the x coordinate and the γH2AX-specific fluorescent signal was determined. The cells were then classified as negative, foci, or pan based on their respective thresholds and split into separate datasets. Next, per mouse, the average number of positive cells was calculated per interval of 500 μm. The mean of all mice within each time point was determined together with the standard deviation (SD). Finally, the graphs were created using the ggplot2 R library by plotting the average relative frequency.

### Statistical analysis

Outliers were identified using the Grubbs test. To test the difference in outcomes between SCI, sham, and naive mice at different time points, a one-way ANOVA was applied (as the naive mice have no overlapping time-points with the sham or SCI groups, a two-way ANOVA was not possible). Normally distributed data (as determined by the Shapiro-Wilk test) was tested with Tukey’s correction for all to all comparisons and Šídák’s correction for more specific hypotheses. When normality assessment was unsatisfactory, non-parametric testing was performed using the Kruskal-Wallis procedure with Dunn’s correction for multiple comparisons.

## Results

### Contusion injury of the spinal cord in mice

**Fig. 1:**
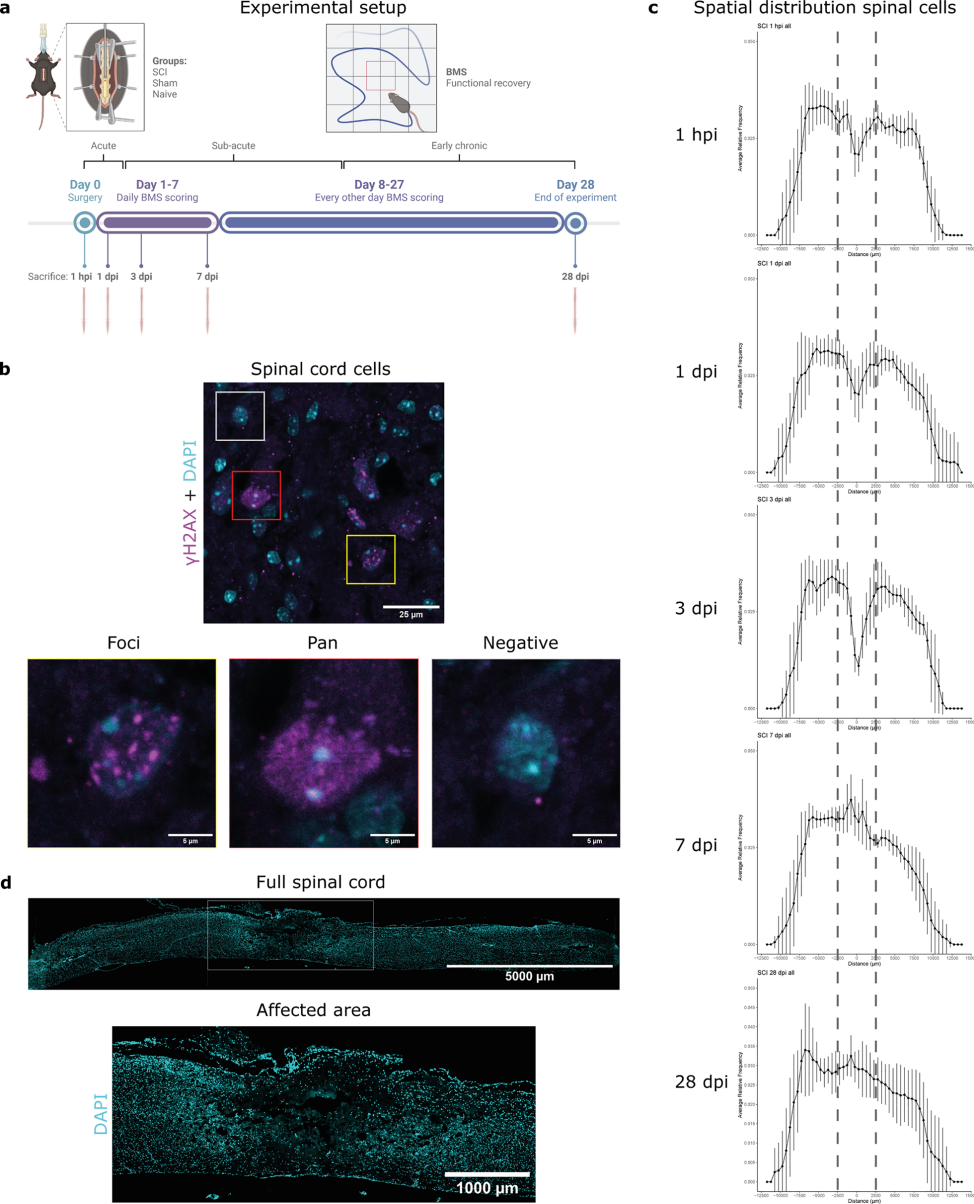
Experimental design of the study. **a** Visual representation of the experimental setup. The mice received, on average, an 82 kdyne contusion injury at L1 of the spinal cord, a sham surgery at L1, or were sacrificed as naive mice. The functional status of the mice was followed up by scoring on the Basso Mouse Scale (BMS). Sacrifice occurred at different time points: 1 h post-injury (hpi), 1 day post-injury (dpi), 3 dpi, 7 dpi, and 28 dpi. **b** The γH2AX intranuclear signal is characterized as foci (yellow box), pan-nuclear (red box), or negative (grey box). Representative images were taken with a confocal microscope (Zeiss, LSM900). **c** Spatial distribution of spinal cord cells at different time points following SCI. The dotted lines show the affected area based on apparent tissue damage and associated cell loss. **d** The affected area is defined by the 5000 μm surrounding the lesion center or homologous sites on sham and naive spinal cords. Error bars represent means ± SD

We used an Infinite Horizon Impactor to induce a spinal cord contusion injury in female C57Bl/6J mice, with an intended 75 kdyne force applied to the center of the L1 spinal level. The degree of force (75 kdyne) was employed to produce a severe injury that causes significant tissue damage but allows for some functional recovery of the mice. Automatic feedback systems of the impactor determined that the mice received, on average, an 82 kdyne contusion at L1 of the spinal cord (Supplementary Fig. 1). All experimental groups (1 hpi, 1 dpi, 3 dpi, 7 dpi, and 28 dpi (Fig. 1a)) had similar starting weights, and all SCI mice received similar impact forces and displacements (Supplementary Fig. 1a-c). The sham mice underwent a laminectomy that resulted in some physical impairment at 1 dpi (not significantly different from naive; *p* = 0.7714), but they recovered to normal locomotion at 2 dpi. Conversely, the SCI mice only slowly and partially regained functionality in the hindlimbs following the SCI, reaching an average BMS score of 2.3 by 28 dpi (Supplementary Fig. 1d).

### Temporal evolution of DNA damage at the affected area of SCI mice

The temporal appearance of DNA damage within the spinal cord following injury was determined by sacrificing mice at several time points post-injury, i.e., 1 hpi, 1 dpi, 3 dpi, 7 dpi, and 28 dpi, and by comparing SCI mice with sham surgery and naive mice (Fig. 1a). Importantly, these time points reflect the different stages following SCI, ranging from acute (0–2 dpi), sub-acute (3–14 dpi), to early chronic phase (from 2 weeks on) of disease progression (Fig. 1a) [2].

We used the well-established DNA DSB marker, γH2AX, to measure a toxic form of DNA damage. Upon DSB formation, ataxia telangiectasia mutated (ATM) kinase is activated and phosphorylates histone 2AX to generate γH2AX (Ser139) as part of a DNA damage response (DDR). γH2AX then recruits, assembles, and activates other factors that execute DNA repair [20]. DNA damage presents as γH2AX foci, which appeared as shown in Fig. 1b, left panel. Pan-nuclear (pan) γH2AX signals are indicative of cell death initiation (Fig. 1b, bottom middle panel) [21]. The analysis of foci and pan γH2AX cells was run automatically based on the aforementioned definitions.

When analyzing the full-length spinal cords for γH2AX labeling (i.e., ~ 1.5 cm in total), we did not find any differences in γH2AX foci and pan signal between the SCI, sham, and naive groups (Supplementary Fig. 2). This likely stems from wash-out of the signal as a result of the inclusion of the large area that does not encompass the injury site or contain damaged tissue and thus has γH2AX presence similar to naive tissue. Thus, to redirect our analysis to the region of the injury, we spatially plotted the DAPI signal of spinal cord cells (Fig. 1c), allowing us to determine the “affected area” unbiasedly. This analysis revealed a lack of or reduced DAPI signal, indicative of cell destruction and loss, that covered 4000 μm at 1 hpi, 5500 μm at 1 dpi, and 6500 μm at 3 dpi (Table 2; Fig. 1c). Thus, we defined the affected area as 2500 μm (2.5 mm) both rostrally and caudally from the center of the lesion (5 mm total), which seems to encompass the most critical impacted area of the induced SCI (Fig. 1d). Notably, at 7 and 28 dpi, there is an apparent filling of the affected area (i.e., increasing DAPI signal), likely explained by infiltration of glia and immune cells at the site of injury [22]. MBP labeling of the SCI tissue revealed that the demyelinated area (traditionally known as the lesion site) caused by the SCI covered a maximum MBP^-^ area length of 1070 μm that is centrally located within the aforementioned affected area (Supplementary Fig. 3). The remaining analysis described here focused on the effects of the SCI on the affected area, which is roughly five times the size of the classically defined lesion site (i.e., 5 mm), as well as the areas along the full-length of the spinal cord.

**Table 2:** Lesion size based on total cell density

Timepoint	Lesion minimum (µm)	Lesion maximum (µm)	Lesion size (µm)
1 hpi	−1250	2750	4000
1 dpi	−2250	3250	5500
3 dpi	−3250	3250	6500
7 dpi	NA	NA	NA
28 dpi	NA	NA	NA

NA, not applicable

**Fig. 2:**
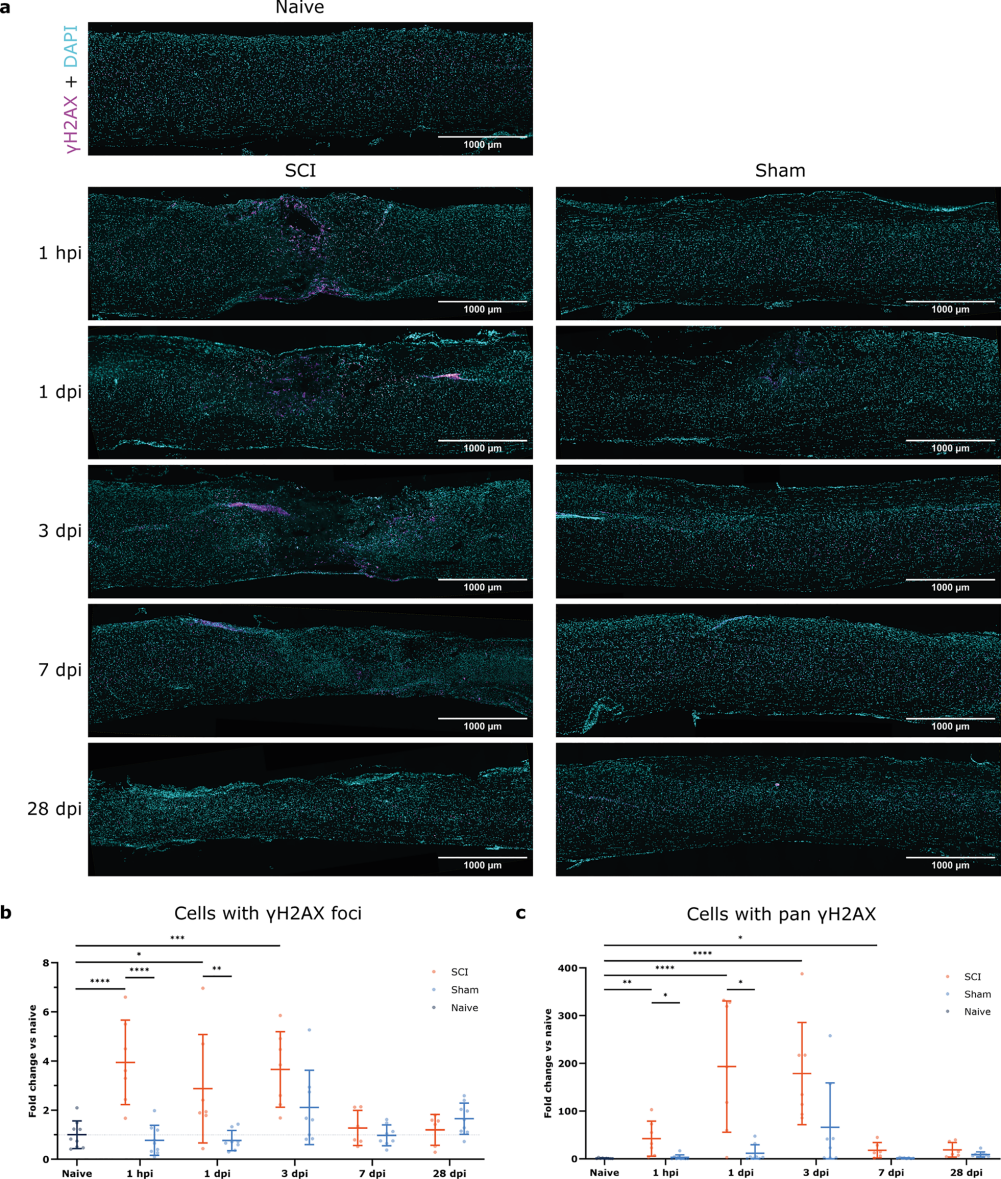
DNA damage occurs at the affected area following SCI from 1 hpi to 3 dpi. Longitudinal spinal cord sections of SCI, sham, and naive mice were labeled for γH2AX (magenta) and DAPI (cyan). **a** Representative images of the affected areas of SCI, sham, and naive spinal tissue at different time points. **b** Timeline of cells with γH2AX foci represented as the fold change of naive mice. Cells with double-stranded DNA breaks are significantly increased in SCI groups at 1 hpi and 1 dpi compared to sham groups and at 1 hpi, 1 dpi, and 3 dpi compared to naive mice. **c** Timeline of cells with pan γH2AX represented as the fold change of naive mice. Cells with pan signal are significantly increased in SCI groups at 1 hpi and 1 dpi compared to sham groups and from 1 hpi up to 7 dpi compared to naive mice. *n* = 6–10. ANOVA with Šídák’s correction for multiple testing was applied. Error bars represent means ± SD. *p < 0.05, **p < 0.01, ***p < 0.001, ****p < 0.0001

With the affected area and injury site established, analysis of the γH2AX focal signals revealed that following SCI, the percentage of cells harboring DNA damage increases locally from 1 hpi until 3 dpi compared to naive mice and from 1 hpi until 1 dpi compared to sham mice. From 7 dpi onward, the DNA damage levels in SCI mice within the affected area return to baseline, as seen in the sham and naive animals (Fig. 2a, b). Surprisingly, the sham group shows a slight increase in DNA damage at the affected area site at 3 dpi, a characteristic that might be explained by inflammatory responses resulting from the laminectomy. Quantification of pan γH2AX signals indicates that early cell death is increased at the SCI affected area from 1 hpi up to 7 dpi compared to naive mice and at 1 hpi and 1 dpi compared to sham mice (Fig. 2c). This pattern expectedly follows the γH2AX DNA damage foci present at earlier time points and then extends to 7 dpi.

Another marker often used for DNA damage, specifically DSBs, is 53BP1. Upon DSB formation, 53BP1 relocalizes to the DNA damage site, where it colocalizes with γH2AX with similar kinetics [23]. When analyzing the immunolabeled images at the affected area (or comparable control sites), we found a decrease in 53BP1-positive cells in SCI samples compared to the sham group at 3 dpi, 7 dpi, and 28 dpi. Moreover, SCI groups (all time points) had, though not significantly, lower 53BP1-positive cells than the naive mice (Supplementary Fig. 4). Consistent with other reports, it would appear that 53BP1 is not a good validation marker for DNA damage or DSBs in at least certain in vivo settings [24, 25].

### Spatial spread of DNA damage in SCI mice over time

**Fig. 3:**
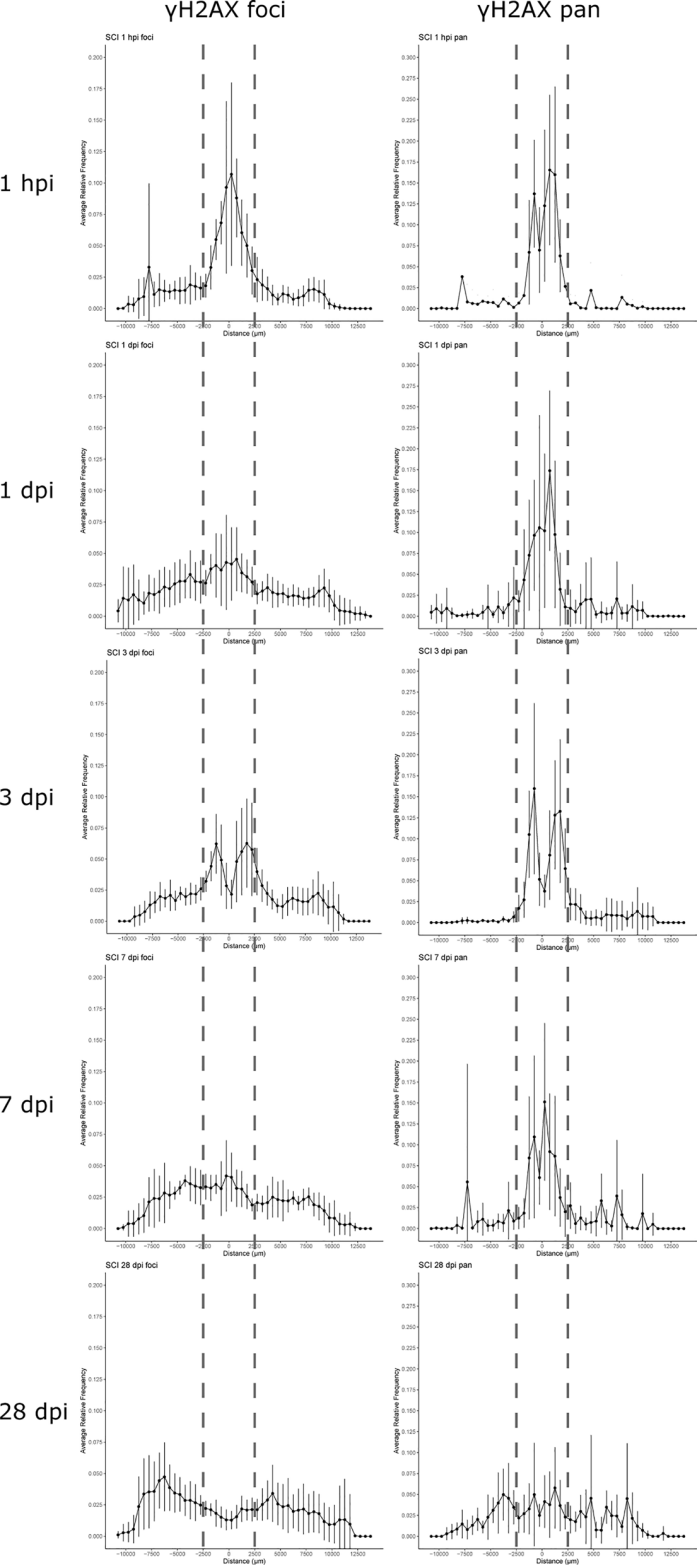
Cells with γH2AX foci spread rostrally and caudally from the lesion center over time following SCI, while cells with γH2AX pan signal remain at the affected area. The dotted lines delineate the affected area (as considered in Fig. 2). The x-axes describe the distance of positive cells from the center of the lesion (0 μm); positive numbers are rostral, and negative numbers are caudal. The y-axes show the average relative frequency (positive cell count per interval over total cell count). Error bars represent means ± SD

Upon visual inspection of Fig. 2a, it was apparent that, over time, the DNA damage signal spreads longitudinally from the lesion center to more rostral and caudal sites that extend beyond the affected area. To quantify this, we determined the distance of γH2AX labeled cells to the defined center of the lesion. We saw that the relative frequency of cells with γH2AX foci increased from 1 hpi to 1 dpi (within the 5 mm affected area) and at 3 dpi started to split into two waves 1750 μm rostrally and 1250 μm caudally from the center of the lesion (Fig. 3; Table 3). At 7 dpi, the DNA damage signal seems to spread further away from the lesion center, and at 28 dpi, the peak signal reaches a distance of 4250 μm rostrally and 6250 μm caudally from the lesion center. These data imply that DNA damage foci start at the injury site but progress along the axis of the spinal cord in both directions (>10 mm total), perhaps with greater speed in the caudal direction compared to rostrally. Looking at the pan cells, we see a peak at 1 hpi until 7 dpi located within the affected area, however, without the further spread seen for γH2AX foci (Fig. 3). At 28 dpi, the pan cells are evenly distributed throughout the entire length of the spinal cord, indicating that early cell death is mainly limited to the affected area.

### DNA damage presence in neuronal cells following SCI

**Fig. 4:**
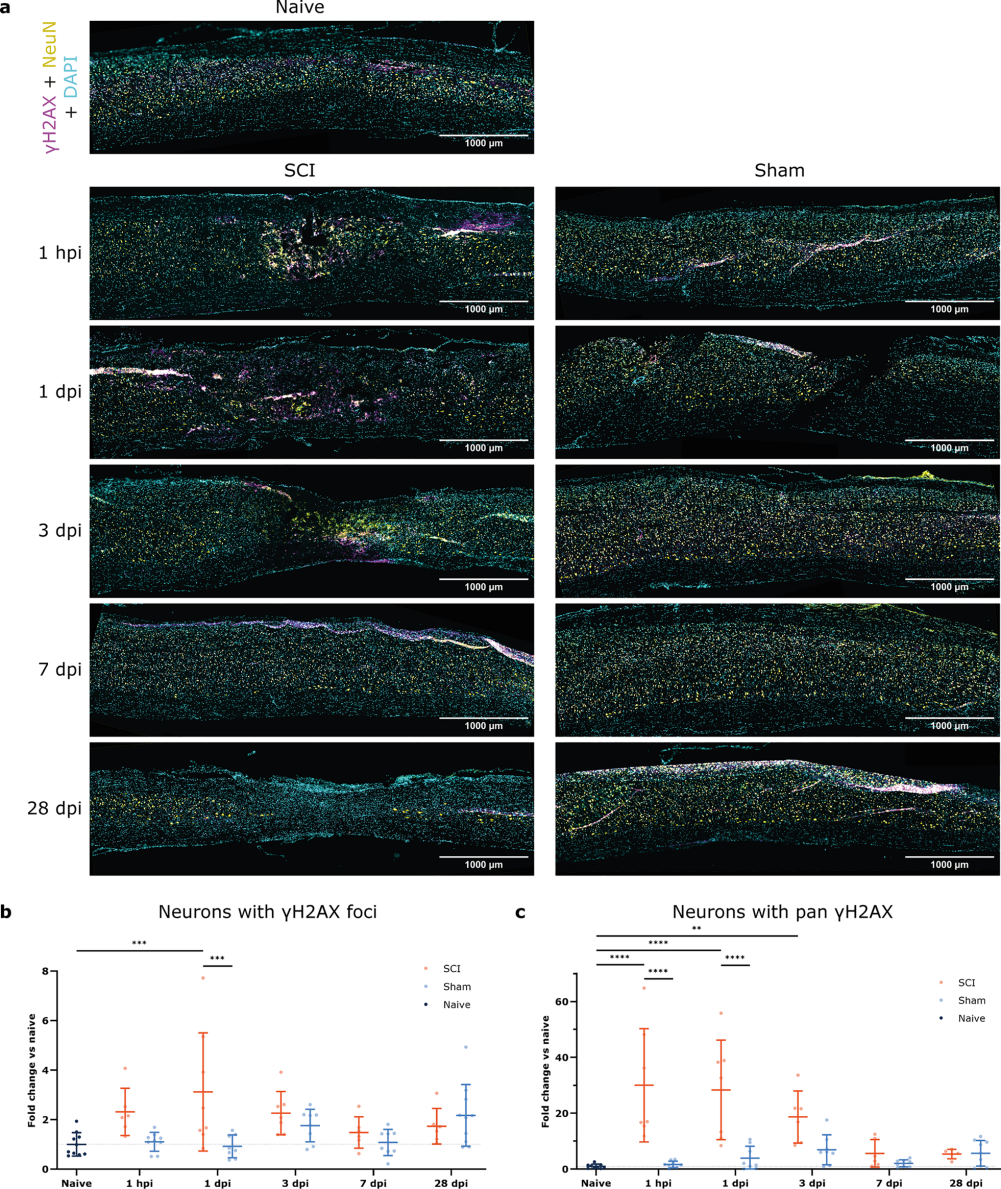
Following SCI, DNA damage is present in neurons at 1 dpi and γH2AX-related early cell death at 1 hpi and 1 dpi. Longitudinal spinal cord sections of SCI, sham, and naive mice were labeled for γH2AX (magenta), NeuN (yellow), and DAPI (cyan). **a** Representative images of the affected areas of SCI, sham, and naive spinal tissue at different time points. **b** Timeline of neurons with γH2AX foci represented as the fold change of naive mice. Neurons with double-stranded DNA breaks are significantly increased in SCI groups at 1 hpi compared to sham and naive mice. **c** Timeline of neurons with γH2AX pan nuclear signal represented as the fold change of naive mice. Neurons with pan signal are significantly increased in SCI groups at 1 hpi and 1 dpi compared to sham groups and at 1 hpi, 1 dpi, and 3 dpi compared to naive mice. ANOVA was applied with Šídák’s correction for multiple testing (**b**) and Kruskal-Wallis test with Dunn’s correction for multiple testing (**c**). *n* = 6–10. Error bars represent means ± SD. **p < 0.01, ***p < 0.001, ****p < 0.0001

To know whether DNA damage contributes to neuronal loss, we determined DNA damage in NeuN-marked neurons explicitly at the affected area. Using a combination labeling of NeuN and γH2AX, we found that neurons with γH2AX foci are more present following SCI at 1 dpi in comparison to sham and naive mice (Fig. 4a, b). Although the temporal DNA damage pattern for neurons is similar to that seen in all cells (Fig. 2b), significance is not achieved for neuronal cells at 1 hpi and 3 dpi. Pan neuronal cells increase at 1 hpi and 1 dpi compared to both sham and naive groups (Fig. 4c). At 3 dpi, an increase in γH2AX pan signal in neurons is only significant compared to the naive mice. Again, the pan pattern mirrors the general pattern of γH2AX in all cells (Fig. 2c).

**Table 3:** Location of γH2AX dense peaks

Timepoint	Caudal peak (µm)	Rostral peak (µm)
**Foci**		
1 hpi	NA	250
1 dpi	NA	750
3 dpi	−1250	1750
7 dpi	−250	
28 dpi	−6250	4250
**Pan**		
1 hpi	−750	750
1 dpi	NA	750
3 dpi	−750	1750
7 dpi	−750	250
28 dpi	NA	1250

NA: not applicable

### Apoptosis

**Fig. 5:**
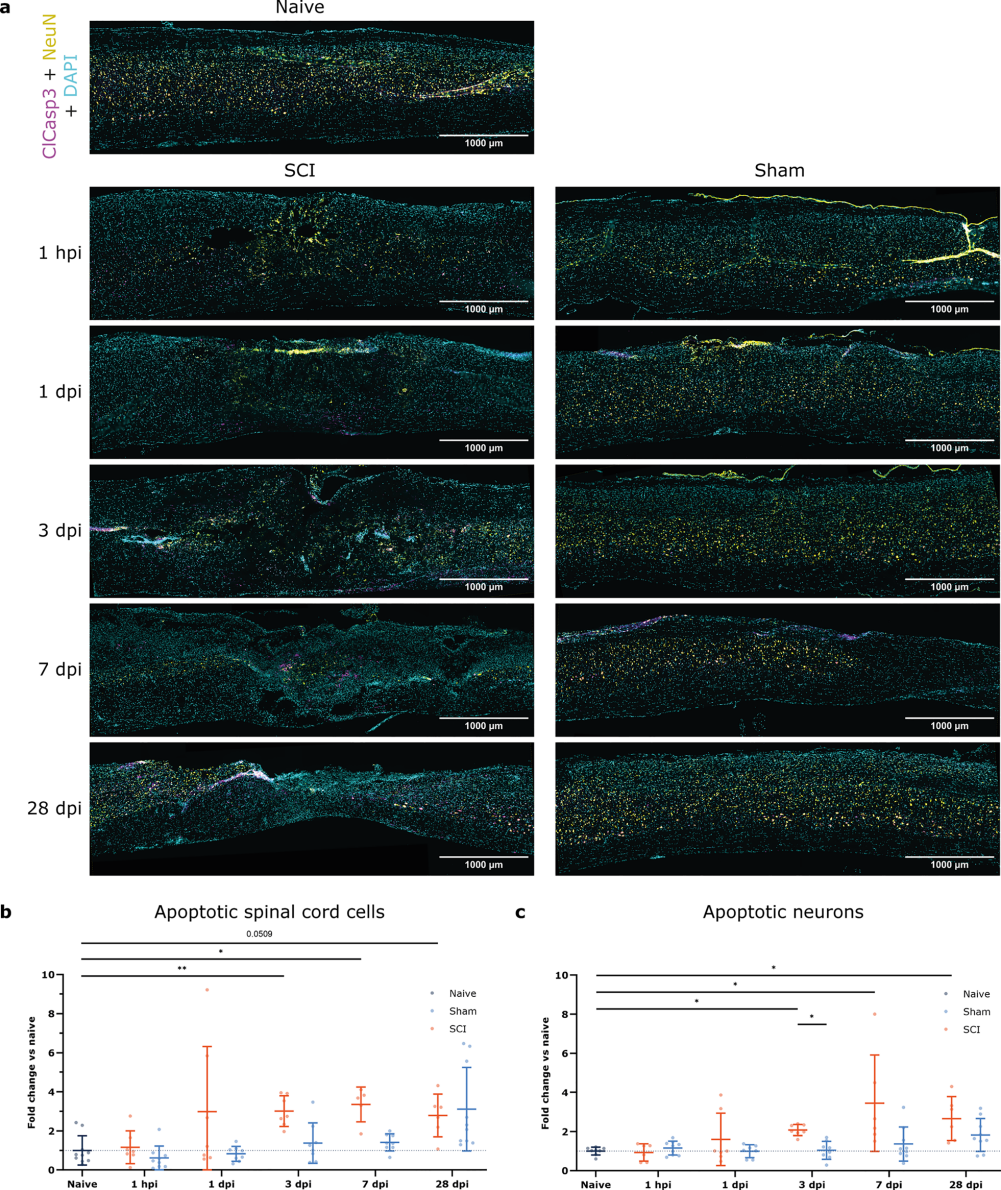
Cellular apoptosis increases following SCI at 3 and 7 dpi vs. naive mice, while neuronal-specific apoptosis is increased from 3 to 28 dpi vs. naive and at 3 dpi vs. sham. Longitudinal spinal cord sections of SCI, sham, and naive mice were labeled for ClCasp3 (magenta), NeuN (yellow), and DAPI (cyan). **a** Representative images of the affected areas of SCI, sham, and naive spinal tissue at different time points. **b** Timeline of all ClCasp3 + spinal cord cells represented as the fold change of naive mice. In the general spinal cord cell population, apoptosis is increased at 3 and 7 dpi compared to naive mice. **c** Timeline of ClCasp3 + neurons represented as the fold change of naive mice. In the neuronal-specific population, SCI increases apoptosis at 3 dpi compared to sham mice and from 3 to 28 dpi compared to naive mice. Kruskal-Wallis test with Dunn’s correction for multiple testing was applied. *n* = 6–10. Error bars represent means ± SD. *p < 0.05, **p < 0.01

As pan γH2AX can be considered an early cell death signal and γH2AX is related to Caspase-dependent apoptosis [21, 26], we wanted to study the relationship of γH2AX and cleaved Caspase-3 (ClCasp3) in the context of SCI. Due to the incompatibility of immunomarkers, γH2AX and ClCasp3 could not be assessed simultaneously. Therefore, we examined for a potential correlation between the two markers when combined with the NeuN marker. Within all spinal cord cells at the affected area, higher ClCasp3-associated apoptosis was found at 3 and 7 dpi compared to naive mice. However, no significant differences existed between SCI and sham-treated groups (Fig. 5a, b). When considering only the neuronal population, the increased ClCasp3 signal compared to naive mice is extended from 3 dpi up to 28 dpi. At 3 dpi, there is also a significant increase in neuronal apoptosis in SCI compared to sham-treated mice (Fig. 5a, c). Comparing the total cell population with either ClCasp3 (onset at 3 dpi) or pan γH2AX signal (onset at 1 hpi), we see that formal apoptosis is detected three days later than pan γH2AX and follows the pan γH2AX up to 7 dpi (Figs. 2c and 5b). The same pattern is seen for the neuronal population, i.e., apoptosis is significant three days later but remains present even at 28 dpi (Figs. 4c and 5c).

## Discussion

When the spinal cord is injured, an aggressive, deteriorating environment is created at the lesion site, leading to primary and secondary neuronal loss within the affected area. Although many pathophysiological processes have been studied and discussed as the source of this loss, effective preventative or regenerative treatments remain elusive. Notably, many of the molecules released after SCI, namely ROS, cause macromolecular damage, particularly in neurons, because of their high metabolic rates and diminished spectrum of DNA repair mechanisms [10]. Thus, as neurons are very susceptible to DNA damage, we hypothesized that neuronal loss might be caused by associated genomic stress and the inability to resolve it.

Previous studies have reported contradicting or inconclusive results regarding DNA damage presence in SCI [16, 27]. While prior reports have mainly indicated increased DNA damage using methods such as HPLC, IHC, WB, ELISA, and Comet assay, the results have varied for several reasons. First, different methods of injury induction have been employed, perhaps indicating that the type of lesion or severity of the damage might affect the degree of genomic stress. Second, the investigations have often lacked comparative controls (sham and/or naive mice). Third, various animal models have been employed, making translation between the potentially pathophysiological processes difficult. Fourth, no cell typing was performed (or reported). Finally, the region of analysis was often not defined, perhaps affecting the results (as seen by our studies using entire spinal cord sections). In the study here, we included sham and naive controls, analyzed the kinetics at key time points, examined region variation, and measured neuron-specificity.

In the γH2AX temporal analysis, we observed increased DNA damage (foci) from 1 hpi to 3 dpi and an apoptotic response (pan) from 1 hpi up to 7 dpi in SCI mice. This early response of γH2AX foci in spinal cord cells of SCI mice relative to sham controls, followed by a more extended persisting pan γH2AX response, is in line with previous kinetic patterns of γH2AX foci and pan signal in other cell models [26]. Our study, therefore, indicates that DNA damage is a very rapid response to a traumatic injury of the spinal cord that persists over several days and likely induces cell death. Notably, an increase of γH2AX was detected in sham-treated mice relative to naive controls, possibly indicating that surgically damaging the peri-spinal environment also affects spinal cord integrity. Ozgonul et al. also reported a slight increase in DNA damage in sham mice in the early chronic phase compared to the acute situation [28]. Apoptosis was increased at 3 and 7 dpi compared to naive mice but not compared to sham mice. Although it is unclear why this occurs, there is speculation regarding the effects of isoflurane on the CNS. Isoflurane appears to be associated with neuroprotection in an injured CNS, while under normal conditions, it seems to act as a neurotoxin [29]. Therefore, the anticipated effects described here could be underestimated, resulting in insignificant differences. In addition, it must be noted that only female mice were used in this study due to technical, ethical, and cost considerations. This limitation should be addressed by replicating the study in a mixed-sex experiment.

One broadly studied mechanism of DNA damage induction is (chronic) inflammation. It has been shown in many different pathological frameworks, e.g., chronic gastritis, chronic lung inflammation, and carcinogenesis, that inflammation can cause and increase DNA damage in other cell types [30–34]. However, external inflammation caused by glia and infiltrating immune cells requires several days to reach the injury site. Therefore, other mechanisms must be in play to cause the early DNA damage response in SCI. Possible early mechanisms are paracrine secretion of ROS, reactive nitrogen species (RNS), excessive glutamate, necrotic signals, or conveyance of signals via gap junctions. Additionally, the acute release of pro-inflammatory cytokines such as TNF-α, IL-1β, and IL-6 may contribute to early DNA damage through inflammatory signaling pathways [35, 36].

As 53BP1 has been reported to operate within the same response pathway as γH2AX [23], we conducted 53BP1 labeling as a verification strategy. However, it was found that 53BP1 labeling increased only in the sham-treated animals relative to both SCI and naive mice. Although 53BP1 labeling has proven reliable in in vitro assays [37, 38], an inconsistent labeling pattern between 53BP1 and γH2AX has been seen in other in vivo studies, particularly those involving mouse pathologic tissue [24, 25]. This fact likely reflects functions for 53BP1 outside of the DNA damage response, such as its role in neurogenesis [39], and points to potential problems with using 53BP1 as a general DNA damage marker.

In the spatial analysis of γH2AX, we found that the DNA damage (γH2AX foci) profile resembles a self-propagating wave that begins at the lesion site and affected area and then flows rostrally and caudally after injury before returning to baseline with time. An explanation could be that cells directly neighboring the primary injury are subjected to a battery of hostile molecules released within the affected area that cause locally induced DNA damage. If these recipient cells subsequently die, the next layer of cells will be exposed to damaging signals. These recipient cells will, in turn, experience consequent genomic stress, and this cycle continues until the response pattern has diminished and returned to normal. A similar longitudinal progression was found for apoptotic cells, as identified by DNA degradation. Crowe et al. showed that apoptotic cells increase over time around the injury site and start bifurcating into two waves at 8 dpi, an observation that complements the pattern of reversible, toxic DNA damage reported herein [15]. Microglia could be one source that contributes to this injury spreading, as they play a major role in both the immediate and delayed phases of secondary injury following SCI [40, 41]. In the acute phase, microglia rapidly activate and release pro-inflammatory mediators. Next, they contribute to oxidative stress, the recruitment of other immune cells, and sustained inflammation. Sub-acutely, microglia maintain the inflammation and participate in other processes such as apoptosis, necrosis, glial scar formation, and potentially chronic neuroinflammation [40, 41].

Another feature of the DNA damage wave is the greater spread and higher presence of DNA damage caudally. This might result from Wallerian degeneration, where the distal segments of the severed spinal axons degenerate, releasing intracellular contents and myelin debris into the extracellular space [42]. This process creates a toxic environment rich in ROS and inflammatory cytokines, which can cause further DNA damage. Additionally, myelin breakdown and microglia activation amplify the local inflammatory response, perpetuating oxidative stress and cytotoxic effects. When considering the early cell death (pan γH2AX cells) profile, the increased signal is restricted to the affected area up to 7 dpi. Therefore, neurons at the affected area seem more prone to early cell death after DNA damage, perhaps due to a more toxic environment as a result of oxidative stress. Contrarily, neurons outside the affected area appear more capable of withstanding DNA damage and mediating survival.

The location of DNA damage following SCI has not been studied before, but a more caudal degradation of the spinal cord has been previously established by Ohnishi et al. [43]. They showed that rostral degeneration is an immediate process exacerbated by oxidative stress, while caudal degeneration is delayed and associated with deficits in the glycolytic pathway. Limited energy supplies have more implications than cell death alone, as DNA repair processes require a lot of energy. Cellular metabolism can, therefore, affect the levels of genomic stress by limiting the available substrates necessary for efficient DNA repair activity [44]. Thus, having compromised energy supplies in the caudal regions of SCI could adversely affect genome maintenance mechanisms, decreasing the capacity of cells experiencing genotoxic threats from resolving induced DNA damage.

Traumatic brain injury (TBI) is the cortical homolog of an SCI. Thus, TBI likely exhibits pathophysiological processes similar to SCI. In TBI, DNA damage, as assessed by e.g., 8-oxoguanine/ γH2AX immunofluorescence or a polymerase I mediated biotin dATP nick-translation assay, has been established as part of the injury pathophysiology in both humans and rodent models [45]. In mouse models involving controlled cortical impact injury, TBI animals exhibited increased DNA damage as early as 15 min post-injury up to 7 dpi [45, 46]. Moreover, γH2AX has even been considered a marker of brain damage [47]. Given our findings, the broader applicability of DNA damage in SCI research requires further investigation.

Focusing on neuronal cells, our experiments showed that DNA damage is present at 1 dpi and that early cell death starts at 1 hpi up to 3 dpi. Above, we note that in the general cell population, elevated levels of DNA damage (γH2AX foci) at 1 hpi and 1 dpi (and 3 dpi vs. naive) precede signs of early cell death (pan γH2AX). In the neuronal population, the DNA damage is limited to 1 dpi. However, it is still proceeded by prolonged elevated early death (pan) markers and apoptosis (ClCasp3) at 3 dpi. This suggests that neuronal cells are more sensitive to cell death following less severe (measurable) levels of DNA damage. This characteristic might be explained by the specific set of DNA repair pathways that neurons possess compared to the broader battery of DNA repair systems available to dividing cells, such as glia. Due to their post-mitotic character, neurons rely primarily on pathways like error-prone non-homologous end-joining (NHEJ), single-strand break repair (SSBR), base excision repair (BER), and nucleotide excision repair (NER). Conversely, major high-fidelity pathways that are intimately connected to DNA replication, such as homologous recombination (HR) and mismatch repair (MMR), are absent in their classic forms [10]. Therefore, neurons might accumulate more DNA damage in a faster timeframe, making them more susceptible to DNA damage-induced cell death, potentially via transcription-blocking neurodegenerative mechanisms [48]. This was shown previously in the retina, where retinal neurons are more sensitive to DNA damage than glia [49]. Therefore, it seems that acute action is needed to avoid DNA damage and associated early cell death to preserve neuronal tissue.

**Fig. 6:**
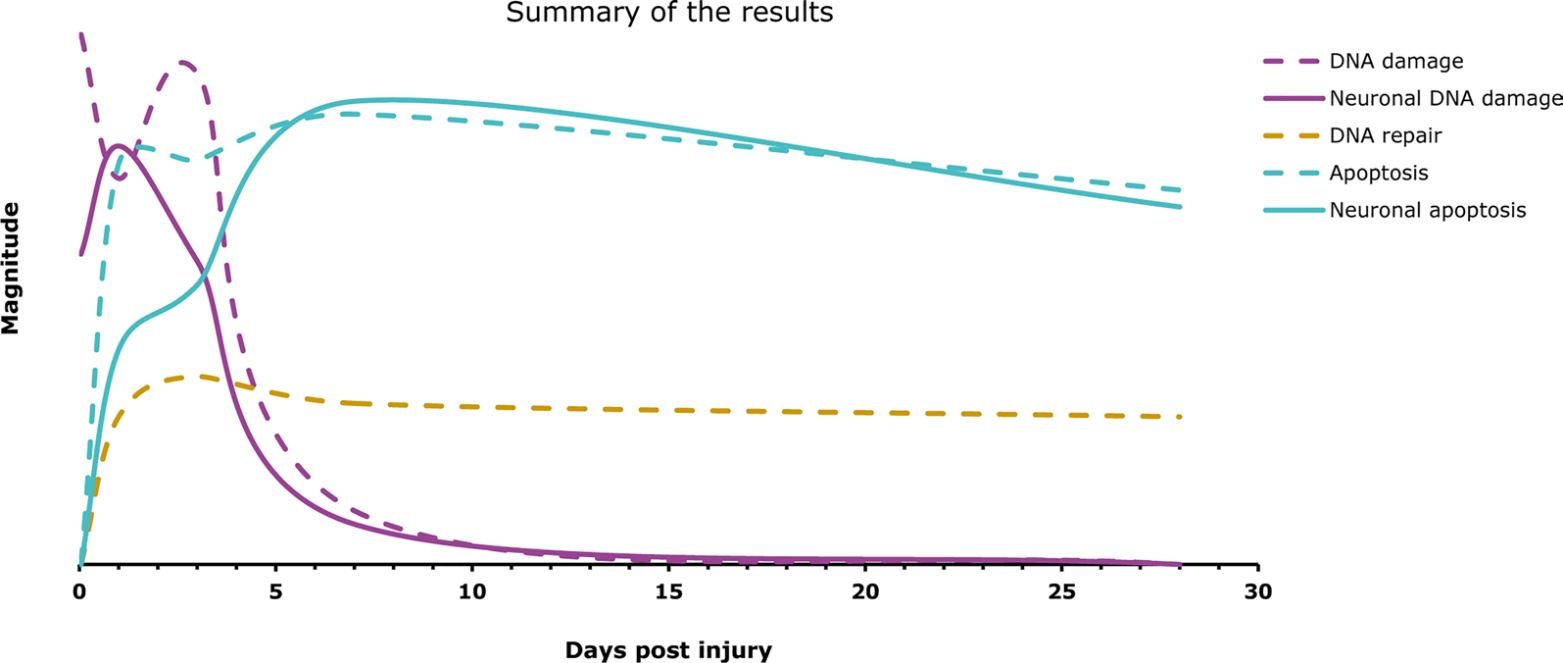
Summary of the results. DNA damage occurs at early time points post-SCI (1 hpi to 3 dpi). DNA repair follows the onset of DNA damage at 1 dpi and remains upregulated up to 28 dpi at least. Apoptosis starts at 1 dpi and remains high until at least 28 dpi. Neuron-specific apoptosis has a slower onset but follows general apoptosis patterns from 7 dpi

Since DNA repair seems inadequate following an SCI, a promising therapeutic tactic would be enhancing DNA repair systems to decrease neuronal loss. In preliminary data mining work investigating DNA repair response pathways, we have seen an upregulation of mechanisms involved in oxidative DNA damage repair with a peak at 3 dpi (unpublished work; Fig. 6). This result correlates nicely with the elevated levels of DNA damage reported at early time points in this paper. Looking to the future, strategies that selectively inactivate hyperactive responses or stimulate underperforming mechanisms could serve as effective therapeutic interventions. One venture targeting the DNA damage response that has shown some success in SCI is Nicotinamide Riboside (NR), a compound that supports poly(ADP-ribose) polymerase (PARP) and strand-break repair mechanisms. A recent study shows that NR increases functional recovery and reduces spinal tissue loss [50], supporting targeting genome maintenance pathways as a therapeutic approach.

## Conclusion

Our study shows, for the first time, that DNA damage rapidly accumulates in the spinal cord following a severe SCI and that there is a spatiotemporal spread of cells harboring DNA damage outward from the affected area. The neuronal population is specifically affected, and although DNA repair mechanisms come into action, DNA damage is still followed by apoptosis (Fig. 6). At present, therapeutic approaches for SCI are limited to surgical removal of debris, corticosteroid treatment, and physical therapy. However, progress is being made in the areas of neuroprotective, neuroregenerative, and neuroprosthetic therapies. Complementary strategies could encompass DNA protection by antioxidants or stimulation of DNA repair mechanisms, potentially minimizing DNA damage-induced cell loss. Future efforts should focus on developing interventions that enhance DNA repair capacity to reduce the cell loss induced by an SCI, thereby preserving spinal cord structure and function.

## Supplementary Information

Supplementary Material 1. The baseline status of the mice was similar among all experimental groups. Mice with a mean weight of 19.8 g ± 1.0received a contusion impact on spinal level L1 with a mean force of 81.7 kdyne ± 6.1and a displacement of the spinal cord tissue of 951 μm ± 221. Functional recovery was assessed using the Basso Mouse Scalescoring. d SCI mice started regaining hind limb function at 7 dpi and reached a mean score of 2.3 ± 1.3 by 28 dpi. The sham mice exhibited mild locomotion deficits at 1 dpi, after which they regained normal locomotion. Kruskal-Wallis test with Dunn’s correction for multiple testingand ANOVA with Tukey’s correction for multiple testingwas applied at α = 0.05. n  = 6–10. Boxplot whiskers represent min-max values

Supplementary Material 2. At full spinal cord length, no increase of DNA damage is present following SCI. Longitudinal spinal cord sections of SCI, sham, and naive mice were labeled for γH2AX and DAPI. a Timeline of cells with γH2AX foci represented as the fold change of naive mice. No significant difference in DNA damage is present when comparing full-length spinal cord tissue of SCI, sham, and naive mice. b Timeline of cells with pan γH2AX represented as the fold change of naive mice. Cells with pan signal are significantly increased in SCI groups at 1 dpi compared to sham groups and at 1 dpi, 3 dpi, and 28 dpi compared to naive mice. Kruskal-Wallis test with Dunn’s correction for multiple testing was applied. n  = 6–10. Error bars represent means ± SD. *p  < 0.05, **p  < 0.01, ****p  < 0.0001

Supplementary Material 3. Demyelinated SCI areas span about 1000 μm, respectively. Longitudinal spinal cord sections of SCI, sham, and naive mice were labeled for MBP (magenta) and DAPI (cyan). a Representative image of SCI spinal tissue at 28 dpi. The grey delineation indicates the MBP - area. b-c MBP - area and length of SCI tissue at different time points. MBP - area ranges from 205 000 µm 2 at 1 dpi to 414 000 µm 2 at 7 dpi ( b ). The length of the demyelinated area ranges from 589 μm at 1 dpi to 1070 μm at 28 dpi ( c ). d The size of the affected area for the analysis of this study was set at 5000 μm. The affected area was defined by the cell density results of Fig. 1; Table 2 and is five times the MBP - area, encompassing the lesion and perilesional area. n  = 3–4. Error bars represent means ± SD

Supplementary Material 4. The number of 53BP1 + cells is increased in sham mice only. Longitudinal spinal cord sections of SCI, sham, and naive mice were labeled for 53BP1 (magenta) and DAPI (cyan). a Representative images of the affected areas of SCI, sham, and naive spinal tissue at different time points. b Timeline of cells with 53BP1 + cells represented as the fold change of naive mice. The number of 53BP1 + cells is significantly increased in sham groups at 1 dpi, 3 dpi, and 28 dpi compared to SCI groups. ANOVA with Šídák’s correction for multiple testing was applied. n  = 6–10. Error bars represent means ± SD. **p  < 0.01, ***p  < 0.001

## Data Availability

Data is housed on a secured UHasselt Google Drive and can be made available to anyone upon request.
