# Supplementary figures and images for: Temporal and spatial pattern of DNA damage in neurons following spinal cord Injury in mice
Source: J Biomed Sci. 2025 Jan 23;32:12. doi: 10.1186/s12929-024-01104-8 (PMC11756142; doi:10.1186/s12929-024-01104-8)

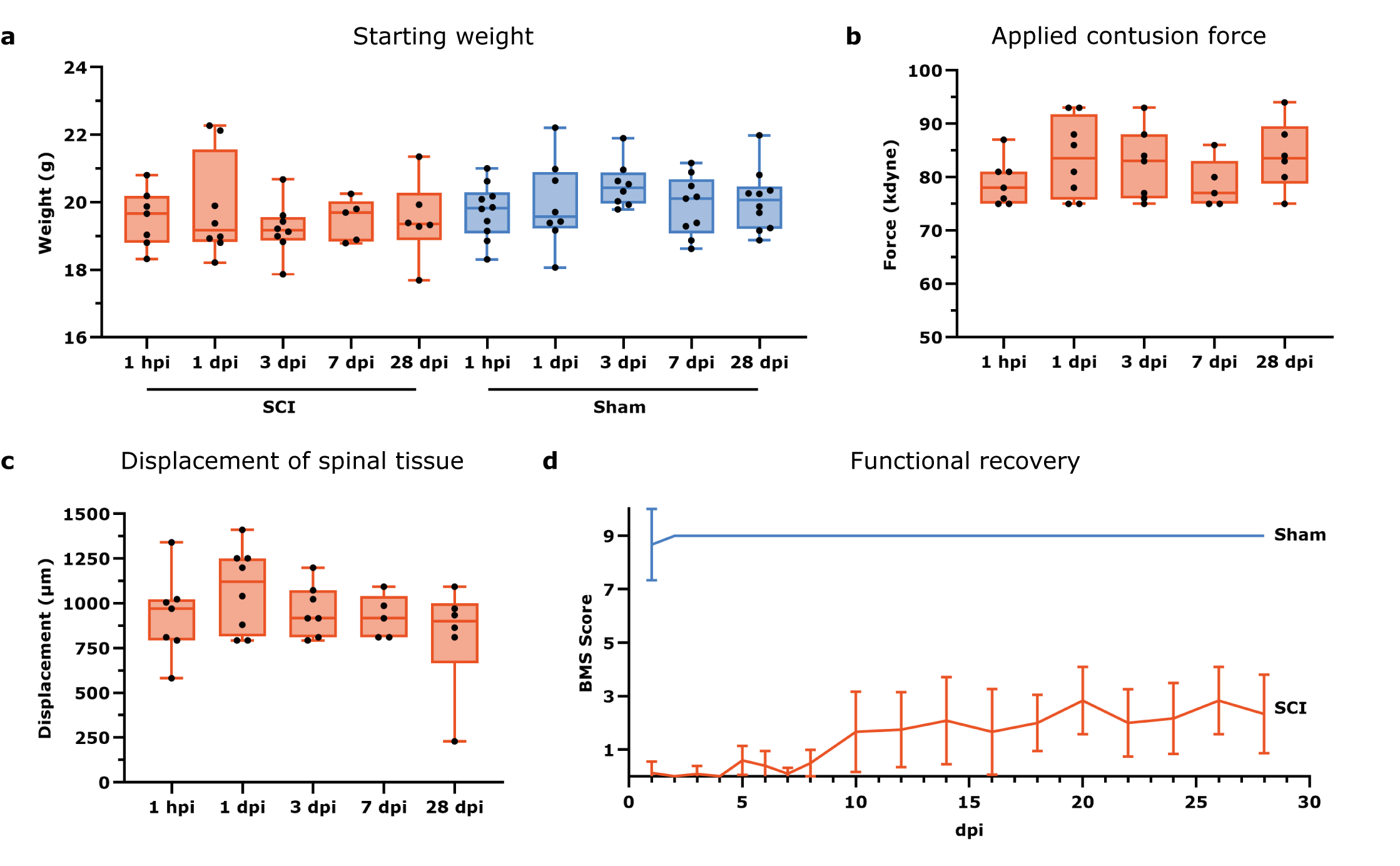

Supplement: Supplementary file 1 — Supplementary Material 1. The baseline status of the mice was similar among all experimental groups. Mice with a mean weight of 19.8 g ± 1.0received a contusion impact on spinal level L1 with a mean force of 81.7 kdyne ± 6.1and a displacement of the spinal cord tissue of 951 μm ± 221. Functional recovery was assessed using the Basso Mouse Scalescoring. d SCI mice started regaining hind limb function at 7 dpi and reached a mean score of 2.3 ± 1.3 by 28 dpi. The sham mice exhibited mild locomotion deficits at 1 dpi, after which they regained normal locomotion. Kruskal-Wallis test with Dunn’s correction for multiple testingand ANOVA with Tukey’s correction for multiple testingwas applied at α = 0.05. n = 6–10. Boxplot whiskers represent min-max values [file 12929_2024_1104_MOESM1_ESM.png]

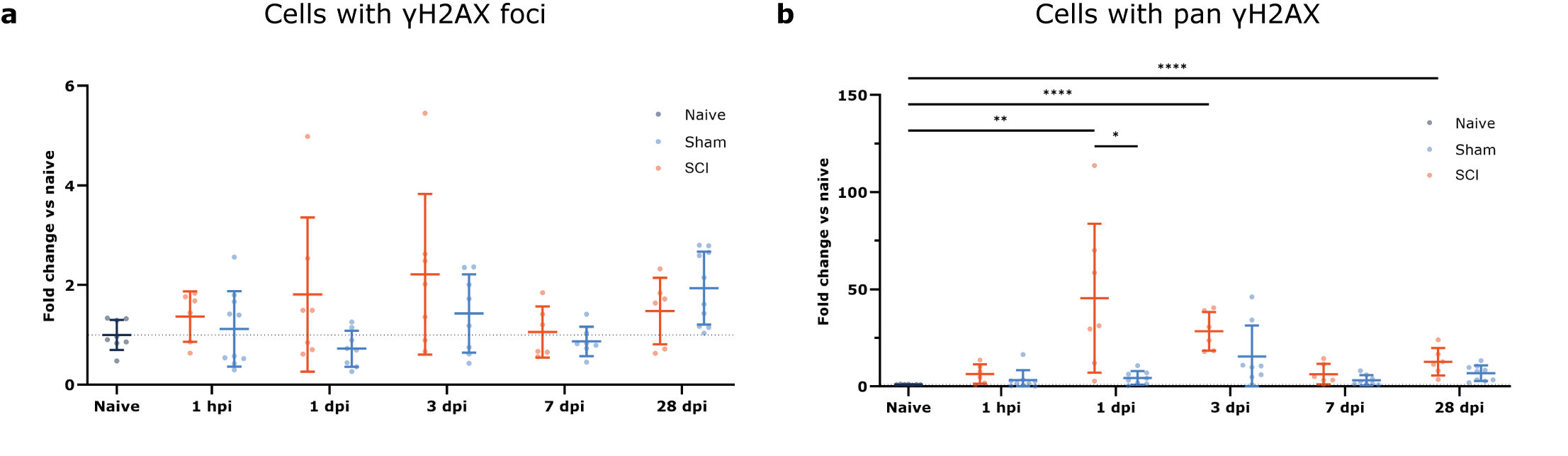

Supplement: Supplementary file 2 — Supplementary Material 2. At full spinal cord length, no increase of DNA damage is present following SCI. Longitudinal spinal cord sections of SCI, sham, and naive mice were labeled for γH2AX and DAPI. a Timeline of cells with γH2AX foci represented as the fold change of naive mice. No significant difference in DNA damage is present when comparing full-length spinal cord tissue of SCI, sham, and naive mice. b Timeline of cells with pan γH2AX represented as the fold change of naive mice. Cells with pan signal are significantly increased in SCI groups at 1 dpi compared to sham groups and at 1 dpi, 3 dpi, and 28 dpi compared to naive mice. Kruskal-Wallis test with Dunn’s correction for multiple testing was applied. n = 6–10. Error bars represent means ± SD. *p < 0.05, **p < 0.01, ****p < 0.0001 [file 12929_2024_1104_MOESM2_ESM.png]

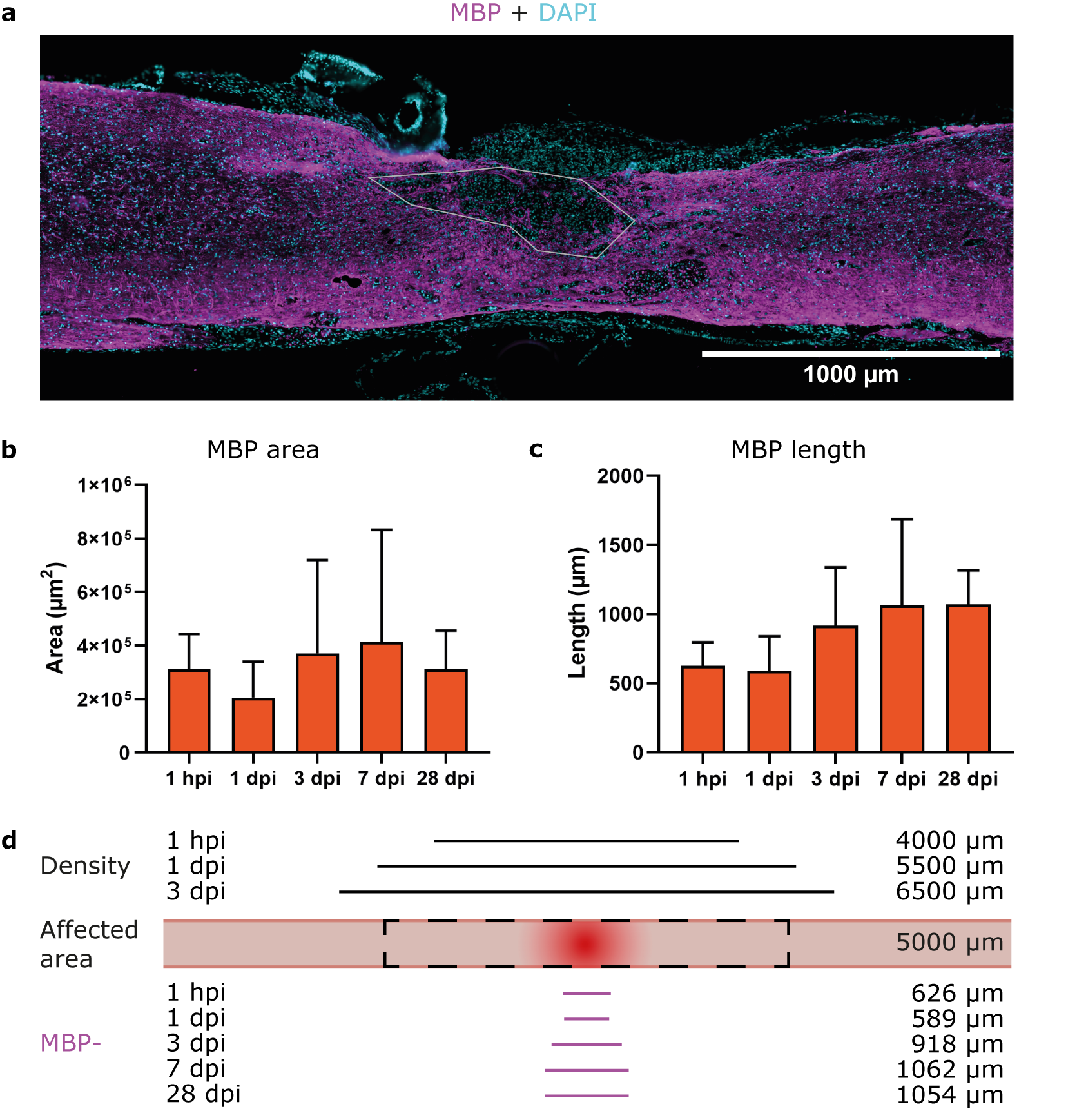

Supplement: Supplementary file 3 — Supplementary Material 3. Demyelinated SCI areas span about 1000 μm, respectively. Longitudinal spinal cord sections of SCI, sham, and naive mice were labeled for MBP (magenta) and DAPI (cyan). a Representative image of SCI spinal tissue at 28 dpi. The grey delineation indicates the MBP - area. b-c MBP - area and length of SCI tissue at different time points. MBP - area ranges from 205 000 µm 2 at 1 dpi to 414 000 µm 2 at 7 dpi ( b ). The length of the demyelinated area ranges from 589 μm at 1 dpi to 1070 μm at 28 dpi ( c ). d The size of the affected area for the analysis of this study was set at 5000 μm. The affected area was defined by the cell density results of Fig. 1; Table 2 and is five times the MBP - area, encompassing the lesion and perilesional area. n = 3–4. Error bars represent means ± SD [file 12929_2024_1104_MOESM3_ESM.png]

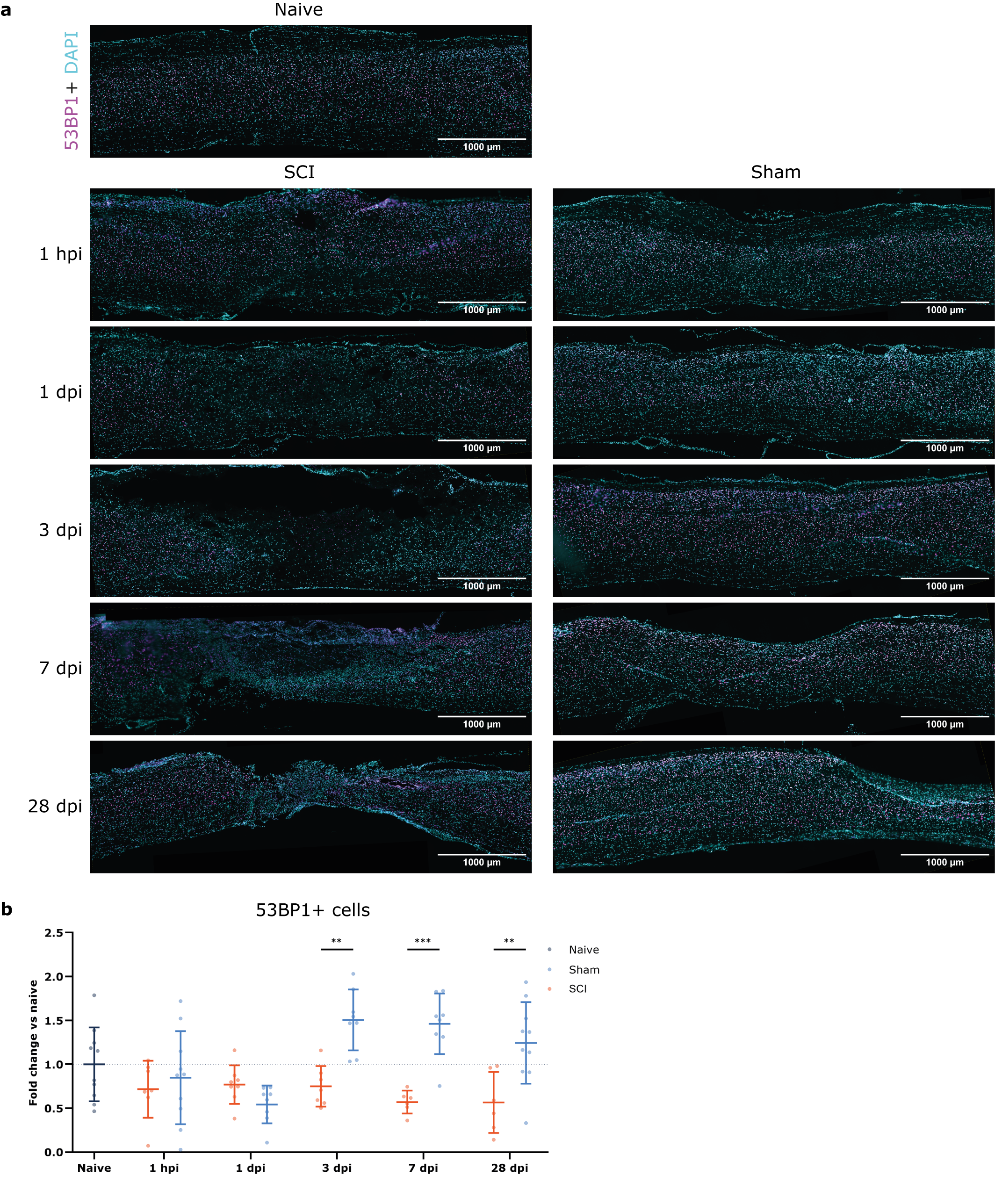

Supplement: Supplementary file 4 — Supplementary Material 4. The number of 53BP1 + cells is increased in sham mice only. Longitudinal spinal cord sections of SCI, sham, and naive mice were labeled for 53BP1 (magenta) and DAPI (cyan). a Representative images of the affected areas of SCI, sham, and naive spinal tissue at different time points. b Timeline of cells with 53BP1 + cells represented as the fold change of naive mice. The number of 53BP1 + cells is significantly increased in sham groups at 1 dpi, 3 dpi, and 28 dpi compared to SCI groups. ANOVA with Šídák’s correction for multiple testing was applied. n = 6–10. Error bars represent means ± SD. **p < 0.01, ***p < 0.001 [file 12929_2024_1104_MOESM4_ESM.png]
